# Venous Thromboembolism Prophylaxis in Hospitalized Medical Patients: A Narrative Review of Risk Stratification, Treatment Duration, and Bleeding Trade-Offs

**DOI:** 10.7759/cureus.117347

**Published:** 2026-10-02

**Authors:** Humza Bari, Fazal Bari

**Affiliations:** 1 OMS-III, University of New England, Portland, USA; 2 Medical Affairs, University of New England, Bernardsville, USA; 3 Oncology, Morristown Medical Center, Morristown, USA

**Keywords:** anticoagulation, dvt, hospital, risk, risk assessment tools

## Abstract

Venous thromboembolism (VTE) remains a leading preventable cause of morbidity and mortality in hospitalized medical patients, yet decisions about who should receive prophylaxis - and for how long - remain far from settled. This review synthesizes evidence from 22 sources, including randomized trials, prospective and retrospective cohorts, systematic reviews, and major society guidelines, addressing risk stratification, in-hospital anticoagulant prophylaxis, extended-duration direct oral anticoagulants (DOACs), the associated bleeding trade-offs, and real-world implementation.

The evidence broadly supports pharmacologic prophylaxis during hospitalization for appropriately selected, acutely ill medical patients with acceptable bleeding risk, with the strongest randomized evidence favoring low-molecular-weight heparin during the inpatient period. That benefit comes with trade-offs worth weighing carefully, since even well-supported regimens carry a measurable increase in bleeding risk alongside their protective effect. Commonly used risk-assessment tools have real limitations as well: the Padua and IMPROVE scores offer a useful clinical framework, but head-to-head comparison shows both discriminate poorly between patients who will and won't develop VTE, and real-world implementation data reveal frequent misclassification and inconsistent dose administration. Extended-duration anticoagulation after discharge follows a similar pattern across the major DOAC trials in this space - fewer thrombotic events, but more bleeding, without a clear survival benefit.

Taken together, this evidence points toward a risk-adapted rather than protocol-driven approach: assess VTE and bleeding risk at admission, initiate appropriate inpatient prophylaxis, reassess as the clinical picture changes, confirm that prescribed doses are actually being given, and reserve extended anticoagulation after discharge for carefully selected patients rather than applying it as a default. Meaningful progress in this area will likely come less from another static risk score and more from models that can track mobility, bleeding risk, and treatment response dynamically over the course of a hospital stay.

## Introduction and background

Venous thromboembolism (VTE) is not a rare event in general medicine - it's one of the more common preventable complications hospitalized patients face. Among whites of European descent, community incidence exceeds one per 1,000 person-years, and VTE carries substantial downstream costs: roughly a quarter of pulmonary embolism cases present as sudden death, and among survivors, about 30% go on to develop recurrence or chronic venous complications within one to two decades [1]. Hospitalization itself is one of the clearest drivers of that risk - one population-based comparison found VTE incidence among hospitalized patients was roughly eight times higher than among community residents during the same period, underscoring that the inpatient stay itself is a distinct, high-risk window rather than simply a setting where community-level risk happens to be observed [2].

Preventing VTE on medical wards is not a simple prescribing task - it's a benefit-versus-harm problem. A single patient might be dealing with acute infection, heart or respiratory failure, cancer, neurologic disease, poor mobility, a prior VTE, and older age all at once, while renal dysfunction, low platelets, active bleeding, recent procedures, or other antithrombotic drugs can make anticoagulating that same patient dangerous. The comparison that matters is not treat versus do nothing - it is weighing a relatively rare but potentially serious event against the bleeding risk of giving a drug to a much larger group of people, most of whom will not develop a clot.

The scale of this problem is not trivial. The ENDORSE study, a multinational cross-sectional survey of over 68,000 hospitalized patients across 32 countries, found that a majority of at-risk medical inpatients did not receive guideline-recommended prophylaxis, highlighting both the prevalence of VTE risk in this population and a persistent gap between guidance and everyday practice [3].

Timing adds another layer. Kolkailah et al.'s 2024 Cochrane review points out that VTE risk sticks around for up to 90 days post-discharge, with most events clustering in the first 45 days, but the review couldn't pin down whether extended treatment is actually worth it net [4]. That splits the clinical question in two: which patients need prophylaxis while admitted, and should a smaller, higher-risk slice of patients keep taking something after they leave.

This review sticks to adult, non-surgical medical inpatients, focusing on how Padua- and IMPROVE-style risk tools get used in practice, low-molecular-weight heparin (LMWH)-based inpatient prophylaxis, and extended direct oral anticoagulant (DOAC) strategies. Randomized and prospective studies carry the most weight for treatment effects; observational work is used more for prediction and implementation questions. Review articles are cited to synthesize findings and flag limitations - not as a substitute for the trials the estimates actually came from.

## Review

Hospitalization as a persistent, not just momentary, risk

Being hospitalized combines immobility with an acute illness that is already prothrombotic. Kolkailah et al. frame VTE risk as something that outlasts the admission itself, and they treat pharmacologic prophylaxis as standard for at-risk acutely ill medical patients without contraindications [4]. The takeaway: admission is where prevention starts, but discharge is not automatically where the biological risk ends.

That does not mean every patient should walk out the door on a blood thinner. It is really the reason extended-duration trials got run in the first place - and why their results have to weigh both late VTE and bleeding side by side. A strategy that cuts down composite or asymptomatic events but raises clinically important bleeding may not actually help an unselected group of medical patients.

Clinical predictors of VTE

Clapham et al.'s 2026 systematic review and meta-analysis flagged cancer, prior VTE, acute infection, diabetes, atrial fibrillation, ischemic stroke, and hypertension as the factors most consistently tied to VTE in hospitalized medical patients [5]. But the same review found moderate-to-serious bias across every included study, and only prior VTE reached moderate-certainty evidence [5]. In other words: structured risk assessment is worth doing, but not every *risk factor* on a checklist deserves equal weight.

Risk also is not fully captured by a diagnosis on a chart. Discharge destination, illness severity, actual mobility, and how the disease evolves day-to-day can all add information a static score misses. A model built only from admission data goes stale fast - the patient who becomes ambulatory, or who deteriorates, has a procedure, or develops new bleeding risk is not the same patient the score described on day one.

Risk versus treatment eligibility

A high probability of VTE does not, by itself, mean anticoagulation is a net win. That call needs a parallel look at bleeding risk, renal function, platelet count, active bleeding, upcoming procedures, and any antiplatelet therapy already on board. Predicting VTE and predicting bleeding are related questions, but they are not the same question, and they should not be answered with the same tool.

Kolkailah et al. found that extended anticoagulation cut short-term symptomatic VTE but increased major bleeding, with essentially no change in all-cause mortality [4]. That is the core tension in one sentence: fewer clots on paper does not necessarily translate into more people surviving if bleeding rises to match.

Practical target of prophylaxis

The goal is not blanket anticoagulation for every admitted patient - it is timely prophylaxis for the ones where expected benefit outweighs expected harm. In practice that breaks into three steps: identify who's at risk, pick an agent and dose that fits their renal function and bleeding profile, and then actually make sure the ordered doses get given.

That third step turns out to matter a lot. Lau et al.'s 2025 retrospective study found only 53.2% of patients got appropriate prophylaxis, and patients who went on to develop in-hospital VTE had more of their prescribed doses skipped than patients who did not [6]. An order that gets held, missed, or refused is not prophylaxis - it is just a note in the chart.

Risk-assessment models in medical inpatients

Padua Approach

The Padua Prediction Score is probably the most widely used tool for gauging VTE risk on medical wards. It was originally derived by Barbar et al. in a prospective cohort of 1,180 hospitalized medical patients followed for up to 90 days, in which a score of 4 or higher identified patients at substantially higher risk of symptomatic VTE, particularly when prophylaxis was withheld [7]. It gives clinicians a structured alternative to gut instinct, prompting them to weigh prior VTE, cancer, immobility, and acute illness systematically. Its real value is as a checklist and a shared decision-making aid - not as a self-justifying reason to anticoagulate on its own.

Drozdinsky et al.'s 2024 retrospective cohort looked at 18,890 acute medical patients before and after mandatory Padua scoring became policy. Prophylaxis use went up once scoring was required, but neither 30-day mortality nor 90-day VTE improved [8]. That is not proof the score itself is flawed - it shows that forcing documentation can change prescribing habits without necessarily improving outcomes if patient selection, adherence, or appropriateness still are not right.

IMPROVE VTE Approach

The IMPROVE VTE model was originally derived by Spyropoulos et al. from the IMPROVE international registry of more than 15,000 acutely ill medical patients, using multivariable analysis to identify independent predictors of VTE both at admission and during hospitalization [9]. It matters most for post-discharge decisions, since the MARINER trial used a modified IMPROVE cutoff - a score of 4 or higher, or 2-3 combined with a D-dimer above twice the local upper limit [10]. That's a good illustration of a broader point: a score built to pick candidates for a 45-day outpatient trial doesn't necessarily work the same way when used to predict hospital-acquired VTE at admission.

That distinction has real consequences. A tool tuned to select a narrow group for an extended outpatient intervention shouldn't be read as a blanket inpatient treatment rule. D-dimer can help enrich for post-discharge risk, but it also just reflects inflammation and illness severity generally, so what it *means* depends heavily on the population and the threshold being used.

Prospective comparison of available scores

Häfliger et al.'s 2024 prospective cohort ran the original and simplified Geneva scores alongside Padua and IMPROVE head-to-head in 1,352 medical inpatients over 90 days [11]. The original Geneva score was first developed by Wicki et al. as a clinical prediction rule to stratify probability of thromboembolic disease using entirely clinical variables [12], and was later streamlined into a simplified version by Le Gal et al. to remove less practical components while preserving predictive performance [13]. Only 28 patients in Häfliger's cohort ended up with symptomatic VTE. The original Geneva score had the best sensitivity; IMPROVE had the best specificity - but all four models performed poorly overall, with AUCs ranging from 53.8% to 58.1% [11].

This carries more weight than a retrospective association because the components were collected prospectively and all models were tested in the same group of patients. The low event count limits how precise the numbers can be, but the message still lands: these scores can organize your thinking without actually being accurate enough to drive highly selective prophylaxis decisions on their own.

Score completion versus score performance

A score can be filled out accurately and still not discriminate well - or it can be statistically solid and still get applied wrong at the bedside. Lau et al. found only 65.3% agreement between the admitting provider's Padua score and a hematologist's independent reassessment; the specialist classified 28.7% of patients as high-risk who'd been marked low-risk on admission [6]. Mobility and acute infection were where agreement broke down most.

Abenante et al.'s 2024 multicenter study found something similar: prophylaxis prescribing tracked with visible features like poor mobility, stroke history, prior clots, or a central line - but the IMPROVE bleeding score didn't seem to factor into the decision much at all [14]. So the gap runs two directions: the models themselves need better predictive accuracy, and clinicians need workflows that actually weigh bleeding risk alongside clotting risk instead of treating a VTE score as a one-way green light to prescribe.

In-hospital pharmacologic prophylaxis

Randomized Evidence for LMWH

The strongest treatment evidence in this whole review comes from Leizorovicz et al.'s randomized, placebo-controlled dalteparin trial. Across 3,706 acutely ill medical patients, 14 days of dalteparin brought the composite VTE outcome down from 4.96% on placebo to 2.77% - an absolute drop of 2.19%, relative risk 0.55. Major bleeding was rare overall but still higher with dalteparin: 0.49% versus 0.16% [15].

That trial makes a solid case for prophylaxis in appropriately selected high-risk inpatients, but it also shows why the benefit-harm math matters. The thrombotic reduction outweighed the bleeding increase in this particular trial population, but that result does not automatically carry over to patients with low baseline risk, active bleeding, severe thrombocytopenia, or a procedure coming up soon.

Duration During the Admission

Inpatient courses tend to run short because the highest-risk window tracks with acute illness and immobility, both of which are temporary by definition. Kolkailah et al.'s synthesis of seven RCTs (40,846 participants total) found that extending anticoagulation past the standard course cut short-term symptomatic VTE but raised major bleeding, with mortality barely moving [4]. That argues for finishing a reasonable inpatient course rather than tacking on extra days just because the patient's still in the hospital.

Duration probably should track the patient's actual trajectory - mobility, illness course, bleeding status - rather than a fixed number of days. Someone who's up and walking and stable looks different, risk-wise, than they did on admission; on the flip side, ongoing immobility or a new infection might justify a second look even if the original score hasn't technically changed.

Agent Selection and Dosing Constraints

LMWH has the strongest direct evidence of anything covered here. Choosing between LMWH, unfractionated heparin, and fondaparinux comes down to renal function, dosing schedule, platelet count, any history of heparin-induced thrombocytopenia, upcoming procedures, and bleeding risk. Nothing in the evidence reviewed here supports swapping standard prophylactic dosing for intensified or intermediate dosing as a default for routine medical inpatients.

Observational comparisons of dose intensity run into confounding by indication pretty easily - doctors tend to give higher doses to patients who already look sicker or riskier, which makes it difficult to attribute any observed outcome difference to the dose itself rather than to the underlying risk that prompted it. Dose escalation is better treated as something that needs patient-specific justification and stronger comparative data from adequately powered trials, not a knee-jerk response to a high score.

When Pharmacologic Prophylaxis Is Unsuitable

Active bleeding, severe thrombocytopenia, an imminent invasive procedure, or another major contraindication shifts the calculation. In those cases, mechanical prevention plus frequent reassessment may be the better route until the contraindication clears. None of the sources here offer a single validated cutoff that can stand in for bedside judgment across every medical population.

That uncertainty is really an argument for documenting why prophylaxis was withheld and when it will be revisited. A one-time assessment at admission does not cut it when the bleeding risk is temporary. And on the other side, continuing anticoagulation without checking for new bleeding or worsening renal function can turn a reasonable earlier decision into a harmful one.

Extended-duration prophylaxis and DOACs

Why Extended Prophylaxis Was Tested

The logic behind extended prophylaxis is straightforward: VTE risk does not vanish at discharge. The complication is that post-discharge event rates are lower than the pooled risk used to enroll many trial participants, while treatment now happens unsupervised - with bleeding, drug interactions, falls, changing renal function, and adherence all becoming live issues.

Snoga et al.'s 2021 review of five RCTs and eight meta-analyses concluded that extended prophylaxis cut VTE at the cost of more major bleeding, with no mortality benefit, and noted that guidelines generally recommend against routinely extending treatment past hospitalization [16]. That points toward selective use, not universal use.

Apixaban After an Acute Medical Admission

In ADOPT, Goldhaber et al. randomized 6,528 medically ill patients to either 30 days of apixaban or 6-14 days of enoxaparin. Among evaluable patients, the primary efficacy outcome occurred in 2.71% on apixaban versus 3.06% on enoxaparin - not a significant difference. Major bleeding, though, was 0.47% versus 0.19%, significantly worse with apixaban [17].

This trial is basically a warning against assuming that oral dosing plus a longer course automatically means better care. Apixaban did not beat the shorter LMWH regimen and caused more major bleeding. Worth noting too: the primary outcome included asymptomatic proximal DVT picked up on screening ultrasound, so it's worth keeping trial-defined efficacy separate from what patients would actually notice or care about.

Rivaroxaban After Discharge

MARINER randomized 12,024 high-risk patients at discharge to either rivaroxaban or placebo for 45 days [10]. The primary composite - symptomatic VTE or VTE-related death - hit 0.83% on rivaroxaban versus 1.10% on placebo, not a statistically significant gap. A prespecified secondary analysis did find less symptomatic nonfatal VTE, while major bleeding was numerically higher on rivaroxaban [10].

The honest read here is narrow: rivaroxaban might help with certain thrombotic outcomes in a carefully enriched group, but the trial did not hit significance on its main outcome. A favorable secondary endpoint is not the same thing as proof that post-discharge treatment should be routine for everyone who crosses a risk-score threshold.

Two Additional Extended-Duration Trials: APEX and MAGELLAN

ADOPT and MARINER are not the only extended-duration DOAC trials in this space. In APEX, Cohen et al. randomized 7,513 acutely ill medical patients with an elevated D-dimer to extended-duration betrixaban (35-42 days) versus standard-duration enoxaparin (10±4 days); the trial found no significant difference in the prespecified primary efficacy outcome, though exploratory analyses in progressively larger, less-selected cohorts suggested a possible benefit for betrixaban [18]. Earlier still, in MAGELLAN, Cohen et al. randomized patients to extended rivaroxaban versus standard-duration enoxaparin and found that rivaroxaban was noninferior for standard-duration prophylaxis and reduced VTE with extended use, but came with an increased risk of clinically relevant bleeding [19].

Comparing eligibility criteria across the four trials makes clear that *high-risk* meant something different in each one. ADOPT enrolled acutely ill medical patients with additional VTE risk factors on top of acute illness alone [17]. MARINER required a modified IMPROVE score of 4 or higher, or a score of 2-3 combined with an elevated D-dimer [10]. APEX enriched specifically for an elevated D-dimer level, evaluated across progressively broader cohorts [18]. MAGELLAN enrolled more broadly, using immobility and other standard acute-illness risk criteria without the D-dimer-based enrichment used in APEX and MARINER [19]. This variation in how each trial defined its *enriched* population is a key reason their results should not be pooled into a single effect estimate, and it is part of why the aggregate signal below should be read as directional rather than as evidence of one uniform effect across agents.

Aggregate Extended-Duration Signal

Kolkailah et al.'s Cochrane review is the broadest synthesis among these sources. Across seven RCTs, extended anticoagulation brought short-term symptomatic VTE down from an estimated 12 to 7 per 1,000 patients, but pushed major bleeding up from 3 to 6 per 1,000, with mortality essentially flat. The certainty rating on these main outcomes was high [4].

That points toward a conservative default. Extended treatment can make sense for carefully chosen patients with genuinely high clot risk and low bleeding risk - but the data does not support extending treatment routinely just because risk technically persists after discharge. Justifying it requires a bigger expected benefit than what an unselected population showed, plus a bleeding risk clearly below the trial average.

Bleeding Risk, Patient Selection, and Net Benefit

A VTE score isn't a bleeding score, full stop. A patient with cancer, a prior clot, and poor mobility can simultaneously have thrombocytopenia, kidney trouble, a recent GI bleed, or dual antiplatelet therapy on board. Treating a high VTE score as an automatic prescription trigger can just mean more anticoagulant exposure without any real improvement in outcomes.

Abenante et al. found that among 1,387 patients with Padua scores of 4 or higher, 66.8% got prophylaxis - but nearly a third of those who also had a lower IMPROVE bleeding score went untreated anyway, suggesting bleeding risk was not really shaping decisions across the board [14]. That's evidence of inconsistent integration between the two scores, not proof that every untreated patient was mismanaged.

Limitations of score-based enrichment

Enriching a population by risk score can increase the absolute benefit of prophylaxis - but only if the score is actually identifying patients whose preventable event rate is high enough to matter. Häfliger et al.'s prospective comparison found weak discrimination across Padua, IMPROVE, and Geneva-based tools [11]. Clapham et al.'s meta-analysis found similar heterogeneity, incomplete risk-factor data, and low-certainty evidence overall [5].

Practically, that means score-based selection needs a clinical assessment layered on top. Crossing a threshold should prompt a closer look at mobility, active infection, cancer status, prior VTE, kidney function, bleeding history, upcoming procedures, and where the patient is headed at discharge. The score provides structure - it does not replace checking whether the inputs are still current or whether treatment is actually safe right now.

Patient selection in extended DOAC trials

Neither MARINER nor ADOPT enrolled a generic ward population. MARINER required either a modified IMPROVE-based profile or a lower score plus an elevated D-dimer [10]. ADOPT enrolled acutely ill medical patients with additional VTE risk factors [17]. APEX specifically enriched for elevated D-dimer [18], and MAGELLAN enrolled broadly acutely ill medical patients using immobility and other standard risk criteria [19]. All four sets of criteria enriched for risk - none guaranteed a favorable net benefit downstream.

The gap between "eligible" and "benefit" shows up clearly in the results. MARINER and APEX both missed significance on their primary efficacy outcomes; ADOPT increased bleeding without improving its primary efficacy outcome; MAGELLAN reduced VTE but at the cost of more clinically relevant bleeding. Entry criteria can successfully find a group with measurable risk while still failing to find a group where extended anticoagulation actually pays off routinely.

Net clinical benefit and patient-important outcomes

The most useful way to rank outcomes puts fatal or disabling clots, major bleeding, and mortality above screen-detected distal or asymptomatic events. The Cochrane review found fewer symptomatic VTE cases but no meaningful mortality difference and more major bleeding with extended treatment [4]. That is a strong argument for spelling out explicitly what outcome a clinician and patient are actually trying to prevent before starting extended treatment.

A decision to extend prophylaxis should lay out the expected benefit, the bleeding risks involved, the planned duration, and what would trigger reassessment. It also needs to account for whether the patient can realistically take the drug correctly once they are home. A relative risk that looks good on paper can still translate into a tiny absolute benefit if baseline event rates are already low.

Implementation, guidelines, and care transitions

Guideline Direction and Evidence Translation

Despite ongoing debate over which risk tool is best, the practical guidance has landed in a fairly stable place, and this consensus has held for some time. As far back as the American College of Chest Physicians' 8th edition guideline, hospitalized medical patients at increased VTE risk were already identified as candidates for pharmacologic prophylaxis [20]. That direction was reaffirmed and refined in the 9th edition, which recommends anticoagulant thromboprophylaxis for acutely ill hospitalized medical patients at increased VTE risk [21], and again in the American Society of Hematology 2018 guideline, which similarly recommends pharmacologic prophylaxis in acutely or critically ill medical inpatients at acceptable bleeding risk while stopping short of endorsing routine extended-duration anticoagulation after discharge [22]. Snoga et al. note that this practical direction is consistent with both bodies' guidance [16].

Admission Assessment Is Not Enough

Lau et al. found frequent disagreement between providers and specialists, with mobility and acute infection among the least consistently assessed variables [6]. Those are exactly the things most likely to change mid-admission. An admission risk score should really be treated as the first data point in an ongoing process, not the final word.

Reassessment makes sense after any major shift - mobility, renal function, platelet count, new bleeding, a procedure, a change in level of care, or a new discharge plan. It is also worth distinguishing a missing score from a genuinely low one, and an intentional decision to withhold prophylaxis from an accidental omission.

Medication Administration Is a Safety Intervention

There is a real difference between prescribing prophylaxis and actually delivering it. In Lau et al.'s cohort, patients who went on to develop VTE had more prescribed doses that were never administered compared to patients who stayed clot-free [6]. That is a case for auditing administration rates, not just counting prescriptions.

A workable pathway should track why each dose gets skipped, minimize unnecessary timing conflicts around procedures, and make renal dose adjustments visible to whoever is giving the medication. It should also flag repeated missed doses for escalation. Electronic alerts can help here, but the evidence points to workflow reliability and someone actually owning the process at the bedside as the real fix.

Discharge as a Treatment Decision Point

Discharge planning should close the loop on whatever prophylaxis was started. The clinician should note whether prophylaxis is stopping because the patient is now mobile and no longer meets an evidence-based indication, continuing because a specific high-risk/low-bleeding strategy was chosen, or changing because a new contraindication showed up.

Nothing in the post-discharge data supports defaulting to a DOAC prescription on the way out the door. Apixaban did not beat the comparator on its primary efficacy outcome and came with more bleeding [17], and rivaroxaban did not hit significance on its primary composite in MARINER either [10]. If extended treatment does get chosen, the indication, duration, renal monitoring plan, bleeding precautions, and who is following up should all be spelled out explicitly.

Research gaps

Predicting Benefit, Not Just Risk

The real unmet need is not another score that predicts VTE generally - it is a model that predicts who actually benefits from prophylaxis. Häfliger et al. found weak discrimination across four validated models [11], and Clapham et al. found the underlying risk-factor literature heterogeneous and mostly low-certainty [5]. Future models should be tested against treatment interaction, not just raw event prediction.

Tracking Mobility and Illness Trajectory as They Change

Mobility matters clinically and gets assessed inconsistently. Lau et al. found poor agreement between routine and expert assessment specifically on mobility [6]. Future studies should use reproducible mobility measures, repeat them through the admission, and test whether tracking change adds predictive value beyond a single admission score.

Outcomes That Actually Matter to Patients

Trials should prioritize symptomatic VTE, major bleeding, clinically relevant non-major bleeding, mortality, and functional outcomes over screen-detected asymptomatic clots. Asymptomatic distal thrombosis might have mechanistic or regulatory value, but it should not drive real-world prevention decisions. MARINER's and APEX's splits between primary and exploratory/secondary results show exactly how much outcome choice can shift the interpretation [10,18].

Safer Strategies After Discharge

The pooled extended-duration evidence across four independent trials and agents shows fewer VTE events, more bleeding, and no clear survival benefit [4]. Future research should push toward narrower patient selection, shorter or adaptive treatment duration, medication adherence, renal monitoring, and outcomes patients actually care about - and should report how many eligible patients can safely finish treatment after discharge, not just how many got randomized (Table 1).

**Table 1:** Summary of key clinical recommendations.

Clinical question	Evidence-based practice point	Key supporting evidence
Should hospitalized medical patients receive pharmacologic VTE prophylaxis?	Yes, for appropriately selected acutely ill patients with acceptable bleeding risk; LMWH has the strongest randomized support for the inpatient period.	[15]
Which risk score should guide patient selection?	Padua and IMPROVE offer a useful structured framework, but neither reliably discriminates who will actually benefit from prophylaxis.	[7,9,11]
Should extended-duration anticoagulation be used routinely after discharge?	No - four independent trials across four different agents consistently show reduced VTE offset by increased bleeding, without a clear mortality benefit in any single trial.	[4,10,17-19]
How reliable is a prescription as a marker of prophylaxis actually being delivered?	Unreliable - roughly half of prescribed doses may not be administered as intended, and missed doses are more common among patients who go on to develop VTE.	[6]
What do current major society guidelines recommend?	Pharmacologic prophylaxis for selected high-risk inpatients without contraindications; no routine extension of anticoagulation past discharge.	[16,21,22]
What should replace a fixed hold interval or one-size-fits-all protocol?	A dynamic, risk-adapted pathway: assess VTE and bleeding risk at admission, reassess as clinical status changes, confirm doses are actually administered, and reserve extended post-discharge treatment for carefully selected patients.	Synthesis of all sources above

## Conclusions

Taken as a whole, this body of evidence resists a simple takeaway. VTE prophylaxis in hospitalized medical patients is not a solved problem waiting for wider adoption - it is a genuine benefit-harm calculation that current tools only partially support. The inpatient period is where the evidence is strongest: short-course LMWH prevents real thrombotic events in appropriately selected, acutely ill patients, a benefit large enough to justify a modest, well-characterized bleeding cost. Everything after discharge is murkier. The four extended-duration DOAC trials reviewed here differed meaningfully in population, agent, dosing duration, and primary endpoint, but each found a similar direction of effect: a reduction in thrombotic events offset by an increase in bleeding, without a clear mortality benefit in any single trial. In the one prospective study that compared them directly in this population, the Padua and IMPROVE risk scores discriminated only modestly better than chance - a finding that should be interpreted within the specific cohort in which it was observed rather than generalized as a fixed property of either score. Perhaps most tellingly, implementation data show that misclassification and missed doses may matter as much as any unresolved question about which drug or which score performs best.

What emerges from all of this is not a protocol but a posture: treat VTE prevention as a dynamic process rather than a one-time admission task. Assess risk and bleeding risk together, choose an evidence-backed inpatient regimen, confirm it is actually reaching the patient, reassess as the clinical picture shifts, and resist the pull toward automatic extension at discharge. The path forward is not another risk score chasing marginally better discrimination - it is models and trials that treat mobility, bleeding risk, and treatment response as things to be measured continuously, and implementation research that takes seriously the finding that the gap between prescribing and delivering may be where the real opportunity for improvement is hiding.

## References

[1] Heit JA (2006). The epidemiology of venous thromboembolism in the community: implications for prevention and management. J Thromb Thrombolysis.

[2] Heit JA, Melton LJ 3rd, Lohse CM, Petterson TM, Silverstein MD, Mohr DN, O'Fallon WM (2001). Incidence of venous thromboembolism in hospitalized patients vs community residents. Mayo Clin Proc.

[3] Cohen AT, Tapson VF, Bergmann JF (2008). Venous thromboembolism risk and prophylaxis in the acute hospital care setting (ENDORSE study): a multinational cross-sectional study. Lancet.

[4] Kolkailah AA, Abdelghaffar B, Elshafeey F (2024). Standard- versus extended-duration anticoagulation for primary venous thromboembolism prophylaxis in acutely ill medical patients. Cochrane Database Syst Rev.

[5] Clapham RE, Speed V, Cox C, Patel JP, Roberts LN (2026). Identifying risk factors for venous thromboembolism in medical inpatients: a systematic review and meta-analysis. Res Pract Thromb Haemost.

[6] Lau BD, Bhave A, Yui JC (2025). Inaccuracies of venous thromboembolism risk assessment and prevention practices among medically ill patients. Blood Adv.

[7] Barbar S, Noventa F, Rossetto V (2010). A risk assessment model for the identification of hospitalized medical patients at risk for venous thromboembolism: the Padua Prediction Score. J Thromb Haemost.

[8] Drozdinsky G, Zusman O, Kushnir S, Leibovici L, Gafter-Gvili A (2024). The effect of obligatory Padua prediction scoring in hospitalized medically ill patients: a retrospective cohort study. PLoS One.

[9] Spyropoulos AC, Anderson FA Jr, FitzGerald G (2011). Predictive and associative models to identify hospitalized medical patients at risk for VTE. Chest.

[10] Spyropoulos AC, Ageno W, Albers GW (2018). Rivaroxaban for thromboprophylaxis after hospitalization for medical illness. N Engl J Med.

[11] Häfliger E, Kopp B, Darbellay Farhoumand P (2024). Risk assessment models for venous thromboembolism in medical inpatients. JAMA Netw Open.

[12] Wicki J, Perneger TV, Junod AF, Bounameaux H, Perrier A (2001). Assessing clinical probability of pulmonary embolism in the emergency ward: a simple score. Arch Intern Med.

[13] Le Gal G, Righini M, Roy PM, Sanchez O, Aujesky D, Bounameaux H, Perrier A (2006). Prediction of pulmonary embolism in the emergency department: the revised Geneva score. Ann Intern Med.

[14] Abenante A, Squizzato A, Bertù L (2025). Predictors for the prescription of pharmacological prophylaxis for venous thromboembolism during hospitalization in Internal Medicine: a sub-analysis of the FADOI-NoTEVole study. Intern Emerg Med.

[15] Leizorovicz A, Cohen AT, Turpie AG, Olsson CG, Vaitkus PT, Goldhaber SZ (2004). Randomized, placebo-controlled trial of dalteparin for the prevention of venous thromboembolism in acutely ill medical patients. Circulation.

[16] Snoga JL, Benitez RM, Kim S, Creager O, Lusk KA (2021). A clinical focus on the use of extended-duration thromboprophylaxis in medically ill patients. Am J Health Syst Pharm.

[17] Goldhaber SZ, Leizorovicz A, Kakkar AK, Haas SK, Merli G, Knabb RM, Weitz JI (2011). Apixaban versus enoxaparin for thromboprophylaxis in medically ill patients. N Engl J Med.

[18] Cohen AT, Harrington RA, Goldhaber SZ (2016). Extended thromboprophylaxis with betrixaban in acutely ill medical patients. N Engl J Med.

[19] Cohen AT, Spiro TE, Büller HR (2013). Rivaroxaban for thromboprophylaxis in acutely ill medical patients. N Engl J Med.

[20] Geerts WH, Bergqvist D, Pineo GF, Heit JA, Samama CM, Lassen MR, Colwell CW (2008). Prevention of venous thromboembolism: American College of Chest Physicians Evidence-Based Clinical Practice Guidelines (8th Edition). Chest.

[21] Kahn SR, Lim W, Dunn AS (2012). Prevention of VTE in nonsurgical patients: Antithrombotic Therapy and Prevention of Thrombosis, 9th ed: American College of Chest Physicians Evidence-Based Clinical Practice Guidelines. Chest.

[22] Schünemann HJ, Cushman M, Burnett AE (2018). American Society of Hematology 2018 guidelines for management of venous thromboembolism: prophylaxis for hospitalized and nonhospitalized medical patients. Blood Adv.

